# Involvement of lncRNA IL21-AS1 in interleukin-2 and T follicular regulatory cell activation in systemic lupus erythematosus

**DOI:** 10.1186/s13075-021-02682-w

**Published:** 2021-12-11

**Authors:** He Hao, Shingo Nakayamada, Naoaki Ohkubo, Kaoru Yamagata, Mingzeng Zhang, Yu Shan, Shigeru Iwata, Tong Zhang, Yoshiya Tanaka

**Affiliations:** 1 First Department of Internal Medicine, School of Medicine, University of Occupational and Environmental Health, Japan, 1-1 Iseigaoka, Yahata-nishi, Kitakyushu, 807-8555 Japan; 2 Department of Internal Medicine, Affiliated Cancer Hospital of Zhengzhou University, Henan Cancer Hospital, Zhengzhou, China; 3 Department of Hematology, The Fourth Hospital of Hebei Medical University, Shijiazhuang, China

**Keywords:** Systemic lupus erythematosus, Lymphocytes, T cells, Molecular biology, Cytokines, inflammatory mediators

## Abstract

**Background:**

The single nucleotide polymorphism (SNP) rs62324212, located in IL21 antisense RNA 1 (IL21-AS1), has been identified as a genetic risk variant associated with systemic lupus erythematosus (SLE). We aimed to probe the characteristics of IL21-AS1 and explore its clinical relevance focusing on T helper subsets and disease activity in patients with SLE.

**Methods:**

rs62324212 genotyping was determined using allelic discrimination by quantitative PCR. Gene expression in peripheral blood mononuclear cells and cell surface markers in CD4^+^ T cells were analyzed using PCR and flow cytometry. The association among IL21-AS1, CD4^+^ T cell subsets, and SLE disease activity was accessed.

**Results:**

Ensembl Genome Browser analysis revealed that rs62324212 (C>A) was located in the predicting enhancer region of IL21-AS1. IL21-AS1 was expressed in the nucleus of CD4^+^ T and B cells, but its expression was decreased in patients with SLE. IL21-AS1 expression was positively correlated with mRNA levels of IL-2 but not IL-21, and it was associated with the proportion of activated T follicular regulatory (Tfr) cells. Furthermore, we observed a significant negative correlation between IL21-AS1 expression and disease activity in patients with SLE (*n* = 53, *p* < 0.05).

**Conclusion:**

IL21-AS1 has an effect on disease activity through an involvement of IL-2-mediated activation of Tfr cells in SLE. Thus, targeting the IL21-AS1 may provide therapeutic approaches for SLE.

**Supplementary Information:**

The online version contains supplementary material available at 10.1186/s13075-021-02682-w.

## Introduction

Systemic lupus erythematosus (SLE) is an immune-related disorder characterized by dysfunctional immune responses, leading to a lack of tolerance to self-antigens and over-secretion of autoantibodies. SLE mainly affects females of reproductive age, and nearly 50% of patients with SLE develop life-threatening complications, including nephritis, pulmonary hypertension, and central nervous system vasculitis [1, 2]. Although an imbalance of CD4^+^ T cell subsets is involved in the pathogenesis of SLE [3], the exact pathogenesis has not been elucidated yet. T follicular helper (Tfh) and T peripheral helper (Tph) cells induce B cell differentiation and autoantibody production. T follicular regulatory (Tfr) cells co-express markers of both T regulatory (Treg) cell and Tfh cell and specifically inhibit co-activation of Tfh cells and B cells to impair antibody maturation and germinal center formation [4]. Recent studies have reported that dysregulation of Tfr cells leads to a variety of autoimmune diseases through the accumulation of autoantibodies. Additionally, in SLE, IL-2 mediates the conversion of Tfh cells to Tfr cells through transcriptional regulation [4]. Therefore, fine-tuning of the imbalance of Tfh and Tfr cells would develop new treatment strategies, such as a low-dose IL-2 therapy, but the underlying mechanisms of this imbalance remain unclear.

A complex interaction of genetic, environmental, and hormonal factors is involved in SLE. Single nucleotide polymorphisms (SNPs) are primarily associated with SLE and other autoimmune diseases by altering the gene function and phenotype, and approximately 90% of SNPs are located in non-coding regions [5, 6]. The genome-wide association studies (GWASs), which apply high-throughput genomic technologies, have identified many susceptible loci [7, 8], and several SLE-associated genes encoded at genetic risk loci, such as *IL-2*, *PDCD1*, *CTLA4*, and *IL-21*, are shown to be involved in the pathogenesis of SLE [9, 10].

The long non-coding RNA (lncRNA), a non-coding RNA containing more than 200 nucleotides, participates in gene regulation in many ways, such as condensing chromatin and chromosome through histone modifications, recruiting transcription factors and polysomes, and altering RNA splicing [11]. LncRNAs participate in gene regulation through both *cis* (near the site of lncRNA) and *trans* (distant site from lncRNA) mechanisms [12]. Recent studies have reported that lncRNAs, such as nuclear paraspeckle assembly transcript 1 (NEAT1), metastasis-associated lung adenocarcinoma transcript 1, and growth arrest-specific transcript 5, are dysregulated in patients with SLE [13]. The abnormal numbers of lncRNAs in the peripheral blood of patients with SLE can be used as potential biomarkers for diagnosis, determining therapeutic responses, and disease prognosis [14].

Interestingly, many disease-associated SNPs are located in the promoter, intron, or exon regions of lncRNAs, suggesting that SNPs may influence the expression levels of lncRNAs or alter their secondary structure, thereby affecting their regulatory functions [15]. Many studies have revealed the correlation between SNPs in lncRNAs and cancer and autoimmune and inflammatory diseases [16–20]. Notably, a study using meta-analysis identified the SNP rs62324212 (C>A), located in IL21 antisense RNA 1 (IL21-AS1), residing just upstream of IL-21 and IL-2 in all ten pediatric autoimmune diseases, including SLE [21]. Moreover, IL-21 is required for the generation of Tfh cells, and IL-2 is necessary for the development of Tfr cells [4, 22]. However, the association among rs62324212, IL21-AS1, and SLE is poorly understood. The current study was designed to assess the characteristics of IL21-AS1 and explore the association among IL21-AS1 expression, cytokines, Th cells, and lupus disease activity.

## Patients and methods

### Patients

The study participants included 53 patients with SLE; 52 patients with rheumatoid arthritis (RA), who were diagnosed according to the American College of Rheumatology revised criteria; and 23 healthy donors (HD). Disease activity was assessed using the Safety of Estrogens in Lupus Erythematosus National Assessment-Systemic Lupus Erythematosus Disease Activity Index (SELENA-SLEDAI). The clinical features of patients are listed in Table 1. The present study was approved by the Institutional Human Ethics Review Committee of the University of Occupational and Environmental Health, Japan, and it was performed according to the 1975 Declaration of Helsinki guidelines. All participants provided written informed consents prior to inclusion in the study. Details that might disclose the identity of subjects have been omitted.

**Table 1:** Demographic and clinical characteristics of study subjects

	SLE (*n* = 53)	RA (*n* = 52)	HD (*n* = 23)
Age (years)	40.9 ± 17.8	64.3 ± 15.7	38.1 ± 8.0
Females, *n* (%)	49 (92.5)	35 (67.3)	20 (87.0)
Disease duration (months)	140.4 ± 159.9	83.6 ± 127.1	
SLEDAI score	4.9 ± 5.4		
BILAG score	4.8 ± 6.6		
Anti-dsDNA (U/ml)	34.4 ± 82.9		
IgG (mg/dl)	1657 ± 710.8	1374 ± 396.3	
CH50 (U/ml)	42.7 ± 14.1		
C3 (mg/dl)	84.7 ± 29.2		
C4 (mg/dl)	16.7 ± 9.1		
CRP (mg/dl)	0.2 ± 0.4	1.5 ± 2.0	
ESR (mm/h)	36.0 ± 31.3	42.0 ± 28.1	
Prednisolone use at baseline, *n* (%)	36 (67.9)	18 (34.6)	
Dose of prednisolone (mg/day)	3.1 ± 3.3	3.8 ± 9.6	
Hydroxychloroquine use at baseline, *n* (%)	30 (56.6)		
Immunosuppressant use at baseline			
Mycophenolate mofetil, *n* (%)	10 (18.9)		
Calcineurin inhibitors, *n* (%)	9 (17.0)		
Azathioprine, *n* (%)	4 (7.5)		
Methotrexate, *n* (%)	2 (3.8)	36 (69.2)	
Mizoribine, *n* (%)	2 (3.8)		
Leflunomide, *n* (%)	1 (1.9)		

Data are mean ± *SD* or percentage of patients

*SLEDAI* Systemic Lupus Erythematosus Disease Activity Index, *BILAG* British Isle Lupus Assessment Group, *Anti-dsDNA* Anti-double-stranded DNA antibody, *CRP* C-reactive protein, *ESR* Erythrocyte sedimentation rate

### Cell isolation

Peripheral blood mononuclear cells (PBMCs) were isolated from blood samples using the lymphocyte separation medium (Cedarlane Corporation) according to the manufacturer’s instructions. Fresh PBMCs were suspended in 100 mL FACS solution (0.5% human albumin and 0.1% NaN_3_ in PBS), and they were stained with the following monoclonal antibodies: anti-CD4-V500 (#560769), anti-CD8-PE-Cy™5.5 (#555368), anti-CD14-PE (#555398), and anti-CD19-FITC (#302206) (BD Biosciences). The stained CD4^+^CD8^−^CD14^−^CD19^-^ T cells, CD4^−^CD8^+^CD14^−^CD19^−^ T cells, CD4^−^CD8^−^CD14^+^CD19^−^ monocytes, and CD4^−^CD8^−^CD14^−^CD19^+^ B cells were incubated at 4 °C for 15 min, and they were sorted using the BD FACSAria II (BD Biosciences). The purity of sorted cells was always > 95%.

### Flow cytometry

Fresh PBMCs from patients and healthy donors were washed and stained with the following antibodies at 4 °C for 15 min: anti-CD3-V450 (#560366, BD Biosciences), anti-CD4-V500 (#560769, BD Biosciences), anti-CD45RA-PE-Cy7 (#560675, BD Biosciences), anti-CXCR5-APC (#356907, BioLegend), and anti-PD-1-PE-Cy7 (#561272, BD Biosciences). For intracellular staining of anti-Foxp3-Alexa Fluor 488 (#560047, BD Biosciences), the cells were fixed and permeabilized with Transcription Factor Buffer at 4 °C for 30 min and were washed with Perm/Wash Buffer (BD Biosciences) before intracellular staining. Isotype-matched control antibodies were used to monitor the background. The well-stained cells were subjected to flow cytometry using a BD FACSLyric system and were further analyzed using the FlowJo v10 software (TOMY Digital Biology).

### Quantitative real-time PCR

Total RNA or cytoplasmic/nuclear RNAs were extracted from cells using the RNeasy Mini Kit (Qiagen) or Cytoplasmic and Nuclear RNA Purification Kit (Norgen), respectively. Subsequently, RNAs were reverse transcribed into cDNA using the High Capacity RNA-to-cDNA kit (Applied Biosystems). Quantitative PCR was performed using a sequence detection system with site-specific primers and probes. The expression level of *GAPDH* was detected as the endogenous control. The primers and probes, *IL21-AS1* (Hs04976181_s1), *IFNG-AS1* (Hs04408308_m1), *NEAT1* (Hs03453535_s1), *IL2* (Hs00174114_m1), *IL21* (Hs00222327_m1), and *GAPDH* (Hs99999905_m1), were purchased from Applied Biosystems. In addition, for the subcellular localization of IL21-AS1, RNA was extracted from the cytoplasmic and nuclear fractions of Jurkat and BJAB cells. Relative mRNA expression levels of *IL21-AS1* and *NEAT1* were evaluated using qPCR.

### Genotyping of SNPs

Genomic DNA was isolated from human PBMCs using the Quick-DNA Miniprep Kit (#D3024, Zymo Research) and quantified using a NanoDrop spectrophotometer (NanoDrop Technologies). The genotypes of rs62324212 were determined using a specific TaqMan SNP genotyping probe (#4351379, Thermo Fisher) and TaqMan™ Universal PCR Master Mix (#4324018, Thermo Fisher Scientific) according to the manufacturer’s protocol, and allelic discrimination was conducted using a quantitative real-time PCR system (Applied Biosystems).

### Statistical analysis

ANOVA was used to compare IL21-AS1 expression among the patients and controls. Differences between the two groups were examined using the unpaired Student’s *t* test or Mann Whitney *U* test. Spearman’s test was used for the correlation analysis between two variables of interest. A *p*-value < 0.05 was considered statistically significant. All statistical tests were performed using the GraphPad Prism software v8 (Prism Software, San Diego, CA, USA).

## Results

### Allele frequencies of rs62324212

The distribution of allele and genotype frequencies of rs62324212 was evaluated among healthy donors and patients with SLE and RA (Table 2). In patients with SLE, the *A* and *C* allele frequencies of rs62324212 were 36.8% and 63.2%, respectively, and the frequencies of the three genotypes were 35.8% (*CC*), 54.7% (*AC*), and 9.5% (*AA*). Despite the small population size, the AA genotype frequencies in patients with SLE were slightly different (not significantly) to those observed in patients with RA (17.4%) and healthy donors (21.7%) and expected using the Ensembl genome database for a Japanese in Tokyo (20.2%) and East Asian population (18.1%). Generally, the minor alleles have led to a high tendency of being risk alleles for complex diseases due to an interplay of multiple genetic and environmental factors [23]. The above results highlight the possibility that the minor allele [A] of rs62324212 may account for genetic susceptibility to SLE.

**Table 2:** Distribution of allele and genotype frequencies for rs62324212 among healthy donors, SLE, and RA patients

	Group	Number	Genotype, *n* (%)			C allele (%)	A allele^a^ (%)
			CC	CA	AA		
Observed	HD	23	6 (26.1%)	12 (52.2%)	5 (21.7%)	52.2%	47.8%
	SLE	53	19 (35.8%)	29 (54.7%)	5 (9.5%)	63.2%	36.8%
	RA	52	15 (28.8%)	28 (53.8%)	9 (17.4%)	56.9%	43.1%
Expected^b^	JPT^c^	104	28 (26.9%)	55 (52.9%)	21 (20.2%)	53.4%	46.6%
	EAS^d^	504	178 (35.3%)	235 (46.6%)	91 (18.1%)	58.6%	41.4%

^a^The allele A belongs to the minor allele

^b^https://asia.ensembl.org/Homo_sapiens/Variation/Population?db=core;r=4:122639284-122640284;v=rs62324212;vdb=variation;vf=72204332#373526_tablePanel

^c^*JPT* Japanese in Tokyo

^d^East Asian

### rs62324212 is located in the enhancer region of IL21-AS1

**Fig. 1:**
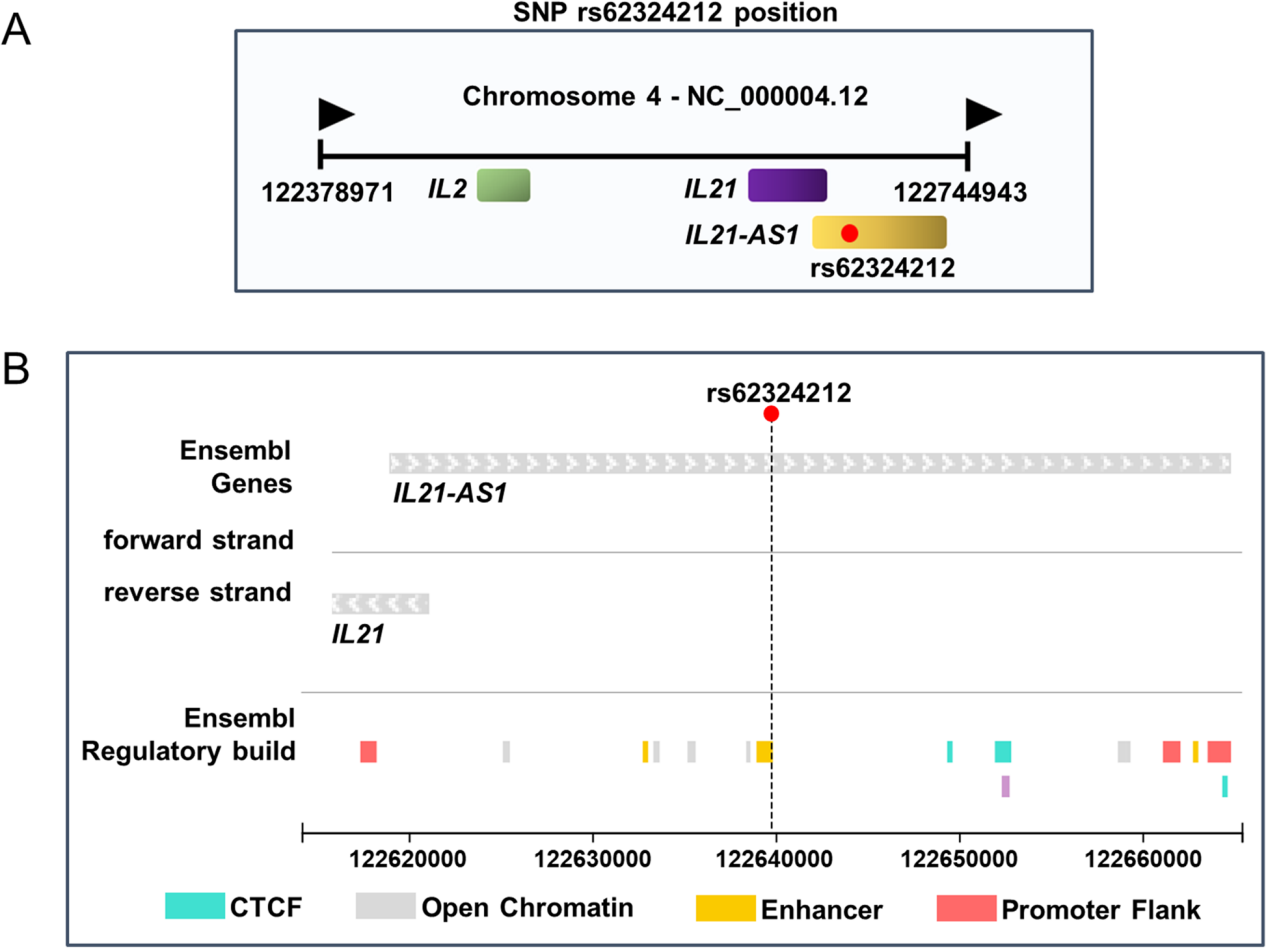
rs62324212 is located in the enhancer region of IL21-AS1 and the minor allele [A] reduces IL21-AS1 expression. **A** Representation of the single nucleotide polymorphism (SNP) rs62324212 (indicated by red dot) and nearby genes. **B** Location of rs62324212 (indicated by dotted line) using the Ensembl Genome Browser. RQ, relative quantity; CTCF, CCCTC-binding factor. Data symbols represent individual subjects; bars denote the mean ± *SEM*. ns, not significant, *p < 0.05, **p < 0.01, ***p < 0.001

We examined the location of rs62324212 in the GWAS Catalog. The SNP rs62324212 was located within the lncRNA IL21-AS1, and the nearby genes were *IL21* and *IL2* (Fig. 1A). The Ensembl Genome Browser analysis suggested that rs62324212 was located in the predicting enhancer region of IL21-AS1 (Fig. 1B). The HaploReg analysis also indicated that rs62324212 resided in an open chromatin region with Histone H3 lysine K4 methylation 1 (H3K4me1) modifications. Since the function of the enhancer is highly cell type-specific, it is important to have information on which cell type it matches with open chromatin. In the HaploReg detail view, we found rs62324212 coincides with the enhancer region of CD4^+^ T cells. The results of multiple bioinformatic tools indicated that rs62324212 might control IL21-AS1 transcription via the regulation of enhancer activity.

### IL21-AS1 is associated with nuclear enrichment

Next, we examined the localization of IL21-AS1 by applying the Ensembl database. IL21-AS1, containing 70,173 bases and located at the chromosome band 4q27, was determined to be a novel antisense gene starting at 123540138 and ending at 123610311. Since the molecular functions of lncRNAs are dependent on proper subcellular localization, we analyzed IL21-AS1 localization in Jurkat and BJAB cells. We found that IL21-AS1 led to nuclear enrichment in Jurkat and BJAB cells, which suggested that IL21-AS1 may play functional roles in the nucleus (Fig. S1).

### Expression of IL21-AS1 is reduced in patients with SLE

**Fig. 2:**
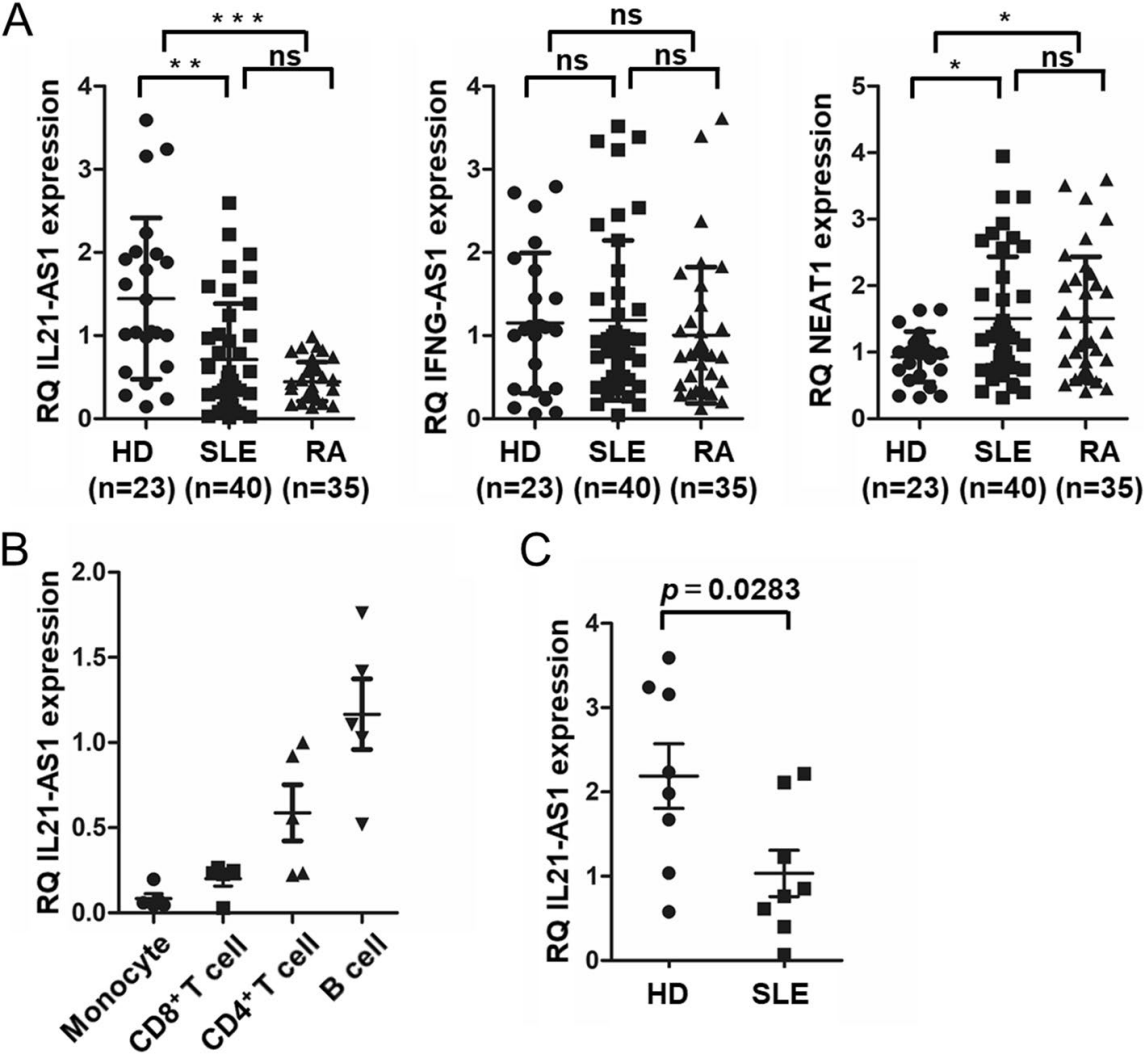
Downregulation of IL21-AS1 in patients with SLE. **A** Relative mRNA expression of *IL21-AS1*, *IFNG-AS1*, and *NEAT1* in isolated PBMCs from patients with SLE (*n* = 40), patients with RA (*n* = 35), and HD (*n* = 23), evaluated using qPCR. **B** Relative mRNA expression of *IL21-AS1* in isolated monocytes, CD8^+^ T cell, CD4^+^ T cell, and B cells from HD (*n* = 5), evaluated using qPCR. **C** Relative mRNA expression of *IL21-AS1* in isolated CD4^+^ T cells from HD (*n* = 8) and patients with SLE (*n* = 8), evaluated using qPCR. Data symbols represent individual subjects; bars denote the mean ± *SEM*. ns, not significant, *p < 0.05, **p < 0.01, ***p < 0.001

We next evaluated the expression levels of three lncRNAs, including IL21-AS1 and IFNG Antisense RNA 1 (IFNG-AS1), in the PBMCs of HD and patients with RA and SLE. The expression of IL21-AS1 in patients with SLE and RA were significantly lower as compared to that in HD (Fig. 2A). The expression of NEAT1 in patients with SLE and RA was higher than that in HD (Fig. 2A). IFNG-AS1 did not show any significant difference among the subjects (Fig. 2A). PBMCs mainly comprised CD4^+^ T cells, CD8^+^ T cells, B cells, and monocytes. We analyzed the expression of IL21-AS1 among the cells. IL21-AS1 levels were higher in CD4^+^ T and B cells relative to those in CD8^+^ T cells and monocytes (Fig. 2B). This result was consistent with the published databases from DICE and GTEx which showed IL21-AS1 was highly expressed in CD4^+^ T cells. Furthermore, IL21-AS1 expression in isolated CD4^+^ T cells was significantly decreased in patients with SLE as compared to that in HD (Fig. 2C).

### IL21-AS1 is associated with the expression of IL-2 and proportion of activated Tfr cells

**Fig. 3:**
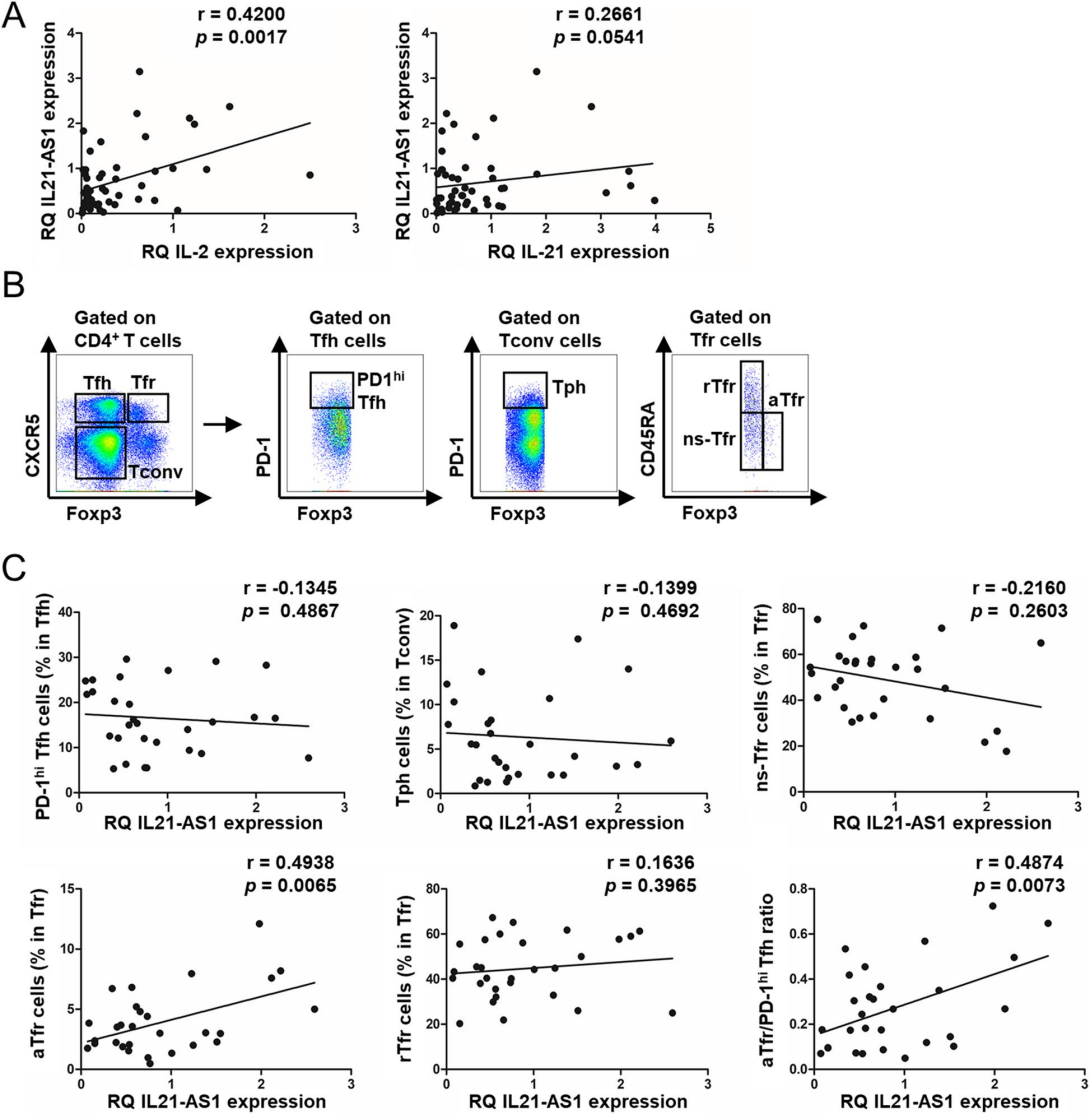
IL21-AS1 is positively associated with IL-2 expression and activated Tfr cells. **A** The relative mRNA expression levels of *IL21-AS1*, *IL2*, and *IL21* were analyzed in the isolated PBMCs of patients with SLE (*n* = 53). Correlation of *IL21-AS1* level with *IL2* and *IL21* in patients with SLE. **B**, **C** PBMCs were isolated from the peripheral blood of patients with SLE (*n* = 29), and they were analyzed using flow cytometry without incubation and qPCR. **B** Gating strategy was employed to identify Tfh (CD4^+^CXCR5^+^Foxp3^−^), Tfr (CD4^+^CXCR5^+^Foxp3^+^), Tconv (CD4^+^CXCR5^−^Foxp3^−^), PD-1^hi^ Tfh (CD4^+^CXCR5^+^Foxp3^-^PD-1^hi^), Tph (CD4^+^CXCR5^-^Foxp3^−^PD-1^hi^), rTfr (CD4^+^CXCR5^+^CD45RA^+^Foxp3^lo^), aTfr (CD4^+^CXCR5^+^CD45RA^−^Foxp3^hi^), and ns-Tfr (CD4^+^CXCR5^+^CD45RA^−^Foxp3^lo^) cells. **C** Correlation of *IL21-AS1* level with the percentage of PD-1^hi^ Tfh cell, Tph cell, Tfr cell subsets, and aTfr/PD-1^hi^ Tfh ratio in patients with SLE (*n* = 29). Data symbols represent individual subjects; Spearman’s test was used for the correlation analysis between two variables of interest

IL-2 and IL-21 are the genes present near IL21-AS1. Therefore, we aimed to investigate whether IL21-AS1 influences IL-2 and IL-21 expression. We evaluated the gene expression levels of IL21-AS1, IL-2, and IL-21 from PBMCs in patients with SLE. IL21-AS1 was positively correlated with the IL-2 gene level, but it did not show any significant correlation with IL-21 (Fig. 3A).

Our recent study demonstrated that an imbalance of Tfr and Tfh cell activation is related to disease activity in patients with SLE, and IL-2 restores the balance between Tfh and Tfr cells through conversion of memory Tfh cells to functional Tfr cells [4]. Therefore, we evaluated whether the observed IL21-AS1 defect in patients with SLE was associated with the proportion of Tfh and Tfr cells. The proportion of Tph cells [24, 25], which are involved in SLE pathogenesis, was also examined. CD4^+^CXCR5^−^Foxp3^−^PD-1^hi^ Tph cells, CD4^+^CXCR5^+^Foxp3^−^PD-1^hi^ Tfh cells, and CD4^+^CXCR5^+^Foxp3^+^ Tfr cells along with their subsets were identified using flow cytometry (Fig. 3B). Both Tph and PD-1^hi^ Tfh cells did not show any significant correlation with IL21-AS1 (Fig. 3C). For the Tfr cell subsets, CD45RA^−^Foxp3^hi^-activated Tfr (aTfr) cells exhibited a positive correlation with IL21-AS1 while CD45RA^−^Foxp3^lo^ non-suppressive Tfr (ns-Tfr) and CD45RA^+^Foxp3^lo^ resting Tfr (rTfr) cells did not (Fig. 3C). Furthermore, IL21-AS1 was positively correlated with the activated Tfr/PD-1^hi^ Tfh ratio (Fig. 3C). Taken together, the above results suggest that IL21-AS1 may be involved in the production of activated Tfr cells.

### Relationship between IL21-AS1 and SLE activity

**Fig. 4:**
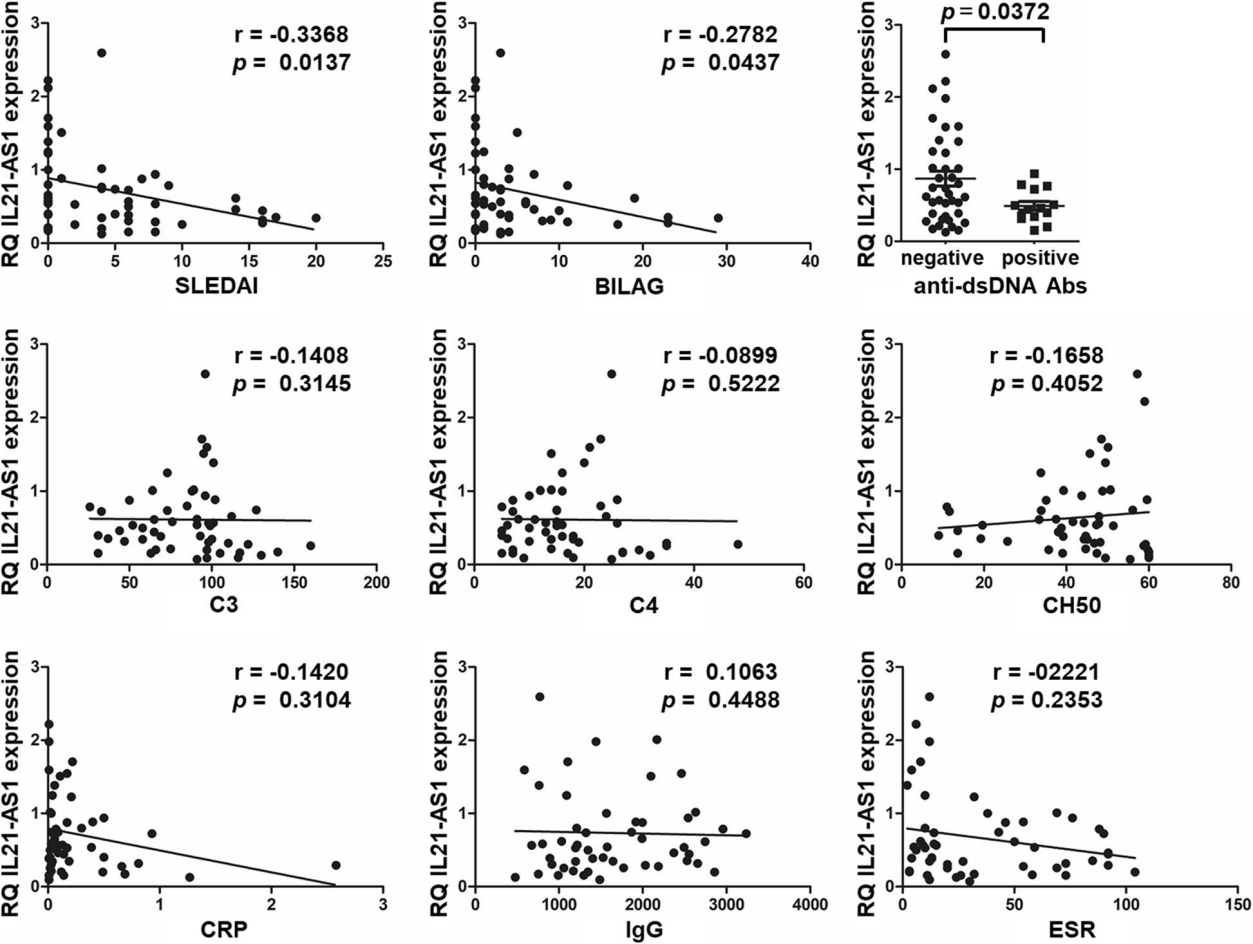
IL21-AS1 is negatively correlated with SLE activity. The relative mRNA expression of IL21-AS1 in isolated PBMCs from patients with SLE (*n* = 53) was evaluated using qPCR. Correlation between IL21-AS1 expression levels and SLE Disease Activity Index, British Isle Lupus Assessment Group, C3 level, C4 level, CH50 level, C-reactive protein (CRP) level, IgG level, erythrocyte sedimentation rate (ESR), and comparisons of IL21-AS1 levels between patients with SLE classified as positive or negative for anti-double-stranded DNA (anti-dsDNA) antibodies. Anti-dsDNA < 12 denotes a negative test (*n* = 39). Data symbols represent individual subjects. Spearman’s test was used for the correlation analysis between two variables of interest

Finally, we explored the correlation of IL21-AS1 with disease activity, serum autoantibody, and inflammation markers in patients with SLE. We found a significant negative correlation between IL21-AS1 expression with SLEDAI or the British Isle Lupus Assessment Group. Besides, IL21-AS1 expression was lower in anti-dsDNA antibody-positive patients than in negative ones (Fig. 4).

## Discussion

After applying a combination of bioinformatic analysis and experimental approaches, we demonstrated frequencies of the minor allele [A] of rs62324212 in patients with SLE and that rs62324212 was located in the enhancer region of IL21-AS1. We demonstrated that the expression of IL21-AS1 was decreased and correlated positively with the expression of IL-2 gene and the proportion of activated Tfr cells in patients with SLE. Furthermore, we found that IL21-AS1 was negatively correlated with disease activity of SLE and anti-dsDNA antibody, suggesting that a decreased expression of IL21-AS1 may promote SLE development. Our previous study [4] showed that the proportion of activated Tfr cells was decreased and negatively correlated with disease activity in patients with SLE and that IL-2 can restore the function of Tfr cells, not only directly expanding the activated Tfr cells but also indirectly converting Tfh to Tfr cells. Those findings indicate that the possible involvement of IL21-AS1 in dysregulated IL-2-Tfr cell axis and then influencing the SLE disease activity.

In this study, we demonstrated that rs62324212 (C>A) was present in the enhancer region, characterized by an open chromatin region with H3K4me1 modification of IL21-AS1, and the minor allele [A] reduced the expression of IL21-AS1. Since IL21-AS1 also decreased in patients with RA, we assessed the correlation between IL21-AS1 and disease activity in patients with RA. However, no significant correlation was observed between IL21-AS1 expression and disease activity scores such as Disease Activity Score with 28 Joint (DAS28) using C-reactive protein (CRP) (DAS28-CRP) and DAS28 using erythrocyte sedimentation rate (ESR) (DAS28-ESR), CRP, ESR, and autoantibodies such as rheumatoid factor and anti-cyclic citrullinated peptide antibody (Fig. S2). Therefore, the function of IL21-AS1 in RA requires further evaluation.

The genetic susceptibility locus of the *IL2/IL21* region at 4q27 has been previously reported in SLE and other autoimmune and inflammatory diseases [26–29]. lncRNAs play regulatory roles through both *cis* and *trans* mechanisms. We found that IL21-AS1 is located near the *IL2/IL21* region, and IL21-AS1 was positively correlated with the *IL2* gene level while IL21-AS1 did not show any significant correlation with *IL21*. Thus, IL21-AS1 may regulate IL-2 production via the *trans* mechanism. However, the detailed mechanism of how IL21-AS1 mediates the generation of IL-2 requires further investigation.

Several studies have noted that lncRNAs are involved in immune cell differentiation and activation, which play an essential role in autoimmune diseases [30–32]. For example, the increased expression of NEAT1, located in the nucleus, contributes to the pathogenesis of SLE [33]. Moreover, many lncRNAs exhibit unique expression profiles in various CD4^+^ T cell subsets, indicating that lncRNAs perform critical roles in CD4^+^ T cell function during disease progression [34–37]. From the Functional Annotation of the Mouse/Mammalian Genome database, we found that IL21-AS1 was highly expressed in normal human lymph nodes, which included a large number of lymphocytes (Fig. S3). From experimental data, we identified that the expression of IL21-AS1 was upregulated in CD4^+^ T and B cells in human peripheral blood. Tfh, Tph, and Tfr cells belong to the CD4^+^ T cell subset, and they play crucial roles in the pathogenesis of SLE [23, 24, 38, 39]. IL21-AS1 showed a significant positive correlation with activated Tfr cells, which were decreased in active patients with SLE due to a defective IL-2 production [4], but not with PD-1^hi^ Tfh and Tph cells. The results of this study along with those of our previous study suggest that IL21-AS1 may contribute to the regulation of IL-2 expression as a part of Tfr cell differentiation and function, providing a possible mechanism for the role of lncRNAs in the regulation of various CD4^+^ T cell subsets.

Our study had some limitations. For instance, according to the Ensembl Genome Browser and HaploReg analyses, rs62324212 is located in the enhancer region of IL21-AS1, but the accurate position still needs to be experimentally confirmed. Whether rs62324212 is directly responsible for the lower IL21-AS1 expression needs to be explored further. It has been reported that lncRNAs are targets of the JAK-STAT signaling pathway during T helper cell differentiation [40]. IL-2-activated STAT3 and STAT5 are critical for Tfr cell differentiation [4]. Although our results indicate that IL21-AS1 is positively correlated with IL-2 levels and proportion of activated Tfr cells, the precise regulatory mechanism for IL21-AS1 and IL-2-Tfr cell axis requires further investigation. Since peripheral T cell subsets may be affected by the treatment of corticosteroids and/or immunosuppressants [41], it is necessary to study patients who are not receiving treatment to eliminate potential effects of treatment on Tfr cell subsets. However, we were not able to obtain a sufficient number of treatment-naive patients in this study. This is a research topic that should be considered in the future.

## Conclusion

In conclusion, our findings indicate the possible involvement of IL21-AS1 in the dysregulated IL-2-Tfr cell axis in patients with SLE. These findings highlight the vital roles of IL21-AS1 during disease progression in patients of SLE. Thus, IL21-AS1 can be treated as a therapeutic target for SLE.

## Supplementary Information

**Additional file 1: Fig. S1.** Subcellular localization of IL21-AS1 in Jurkat and BJAB cells. RNA was extracted from the cytoplasmic and nuclear fractions of Jurkat and BJAB cells. Relative mRNA expression levels of *IL21-AS1* and *NEAT1* were evaluated using qPCR.

**Additional file 2: Fig. S2.** Correlation between IL21-AS1 and disease activity of RA. The relative mRNA expression of IL21-AS1 in isolated PBMCs from patients with RA (*n* = 44) was evaluated using qPCR. Correlation between IL21-AS1 expression levels and RA Disease Activity Score with 28 joint (DAS28) using CRP (DAS28-CRP) and DAS28 using ESR (DAS28-ESR), CRP level, ESR level, rheumatoid factor level, and anti-cyclic citrullinated peptide antibody level. Data symbols represent individual subjects. Spearman’s test was used for correlation analysis between two variables of interest.

**Additional file 3: Fig. S3.** Expression level of IL21-AS1 in human normal tissues. Expression level of IL21-AS1 was obtained from the Functional Annotation of the Mouse/Mammalian Genome database. CPM: counts per million.

## Data Availability

The datasets used and/or analyzed during the current study are available from the corresponding author on reasonable request.

## Abbreviations

IL-2: Interleukin-2
SNP: Single nucleotide polymorphism
IL21-AS1: IL21 antisense RNA 1
SLE: Systemic lupus erythematosus
Tfr: T follicular regulatory
Tfh: T follicular helper
Tph: T peripheral helper
GWASs: Genome-wide association studies
lncRNA: Long non-coding RNA
NEAT1: Nuclear paraspeckle assembly transcript 1
RA: Rheumatoid arthritis
HD: Healthy donors
PBMCs: Peripheral blood mononuclear cells
H3K4me1: Histone H3 lysine K4 methylation 1
IFNG-AS1: IFNG antisense RNA 1
SELENA-SLEDAI: Safety of Estrogens in Lupus Erythematosus National Assessment-Systemic Lupus Erythematosus Disease Activity Index
DAS28: Disease Activity Score with 28 Joint
CRP: C-reactive protein
ESR: Erythrocyte sedimentation rate
