# Supplementary figures and images for: Involvement of lncRNA IL21-AS1 in interleukin-2 and T follicular regulatory cell activation in systemic lupus erythematosus
Source: Arthritis Res Ther. 2021 Dec 11;23:302. doi: 10.1186/s13075-021-02682-w (PMC8665514; doi:10.1186/s13075-021-02682-w)

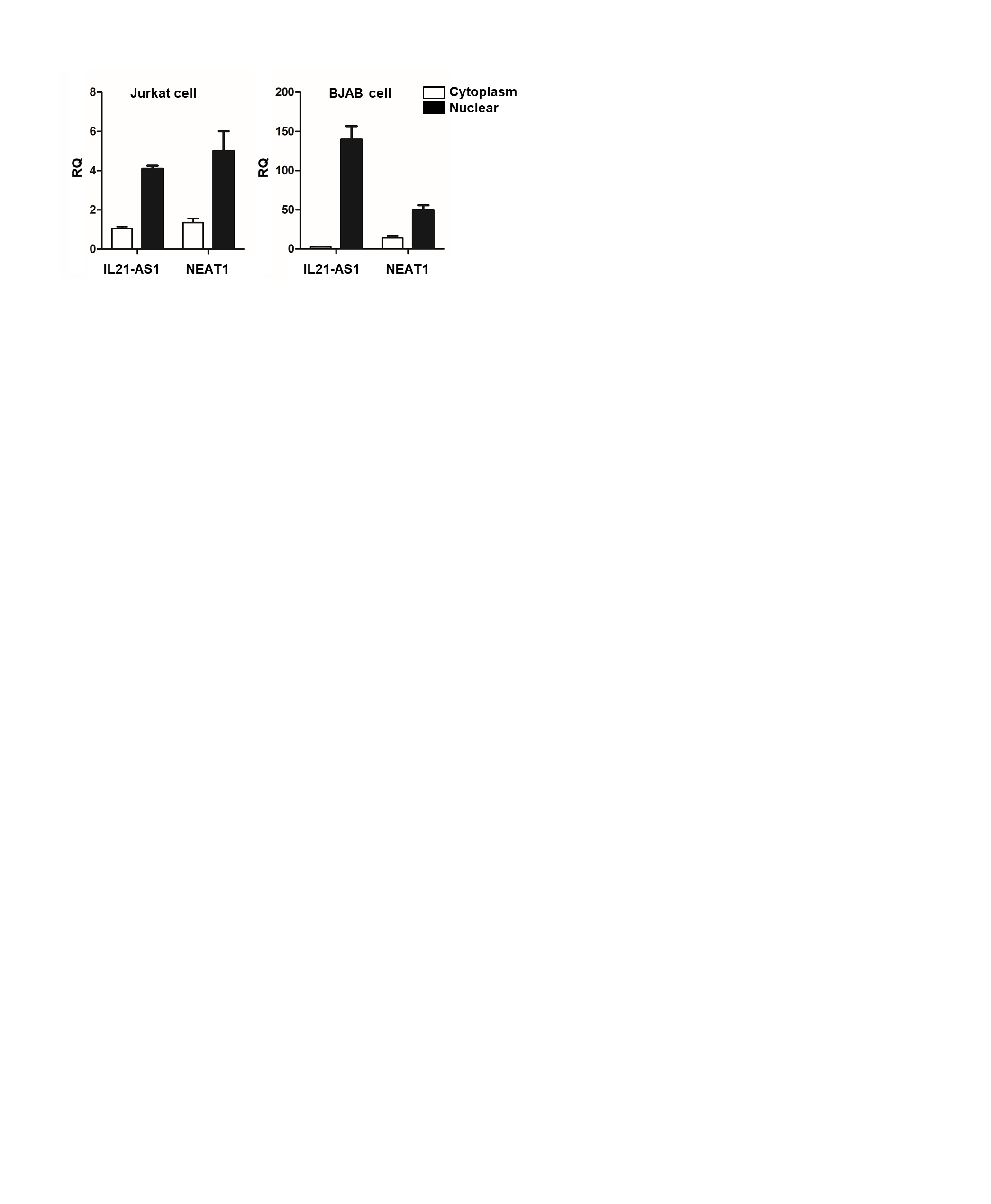

Supplement: Supplementary file 1 — Additional file 1: Fig. S1. Subcellular localization of IL21-AS1 in Jurkat and BJAB cells. RNA was extracted from the cytoplasmic and nuclear fractions of Jurkat and BJAB cells. Relative mRNA expression levels of IL21-AS1 and NEAT1 were evaluated using qPCR. [file 13075_2021_2682_MOESM1_ESM.tif]

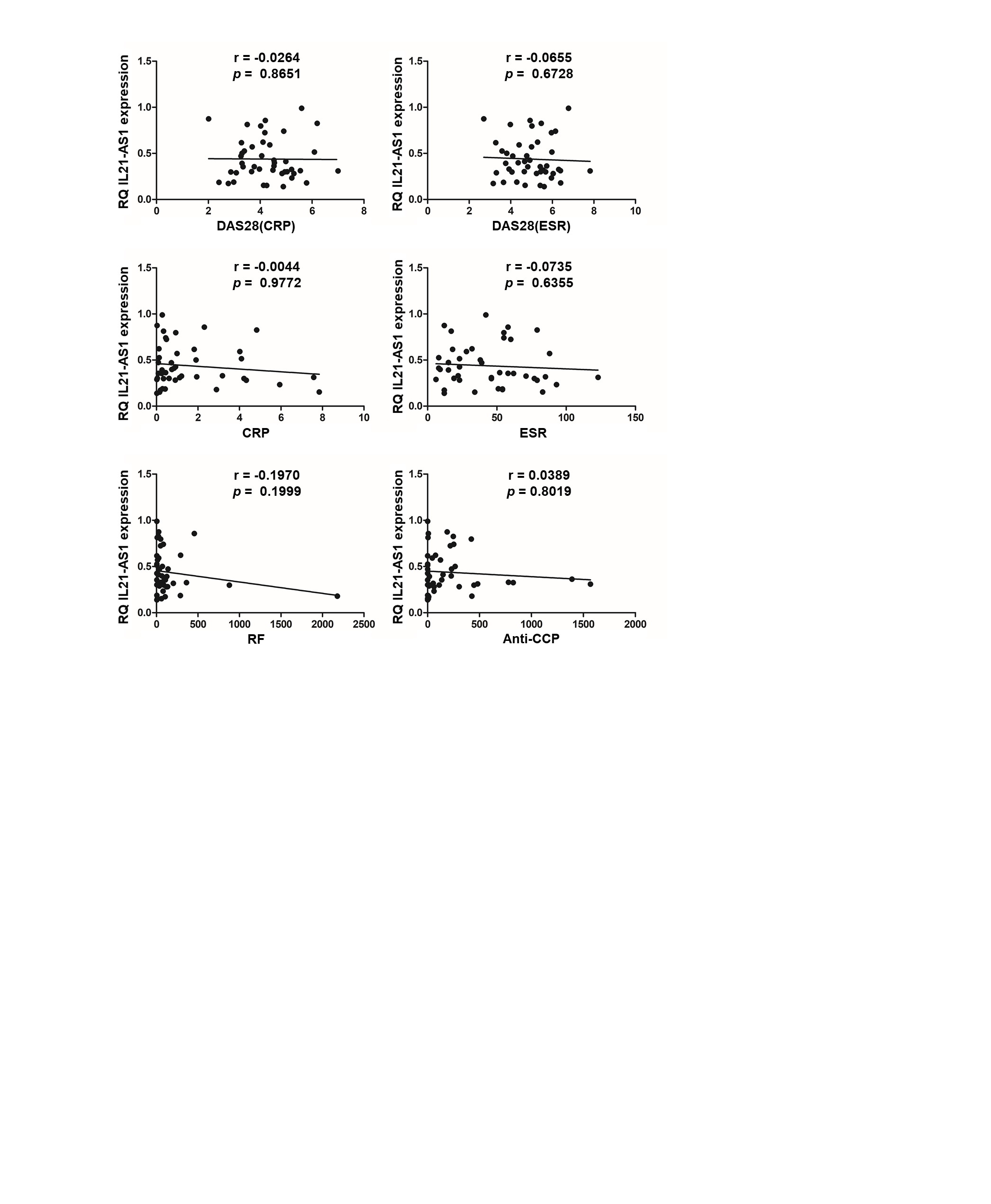

Supplement: Supplementary file 2 — Additional file 2: Fig. S2. Correlation between IL21-AS1 and disease activity of RA. The relative mRNA expression of IL21-AS1 in isolated PBMCs from patients with RA (n = 44) was evaluated using qPCR. Correlation between IL21-AS1 expression levels and RA Disease Activity Score with 28 joint (DAS28) using CRP (DAS28-CRP) and DAS28 using ESR (DAS28-ESR), CRP level, ESR level, rheumatoid factor level, and anti-cyclic citrullinated peptide antibody level. Data symbols represent individual subjects. Spearman’s test was used for correlation analysis between two variables of interest. [file 13075_2021_2682_MOESM2_ESM.tif]

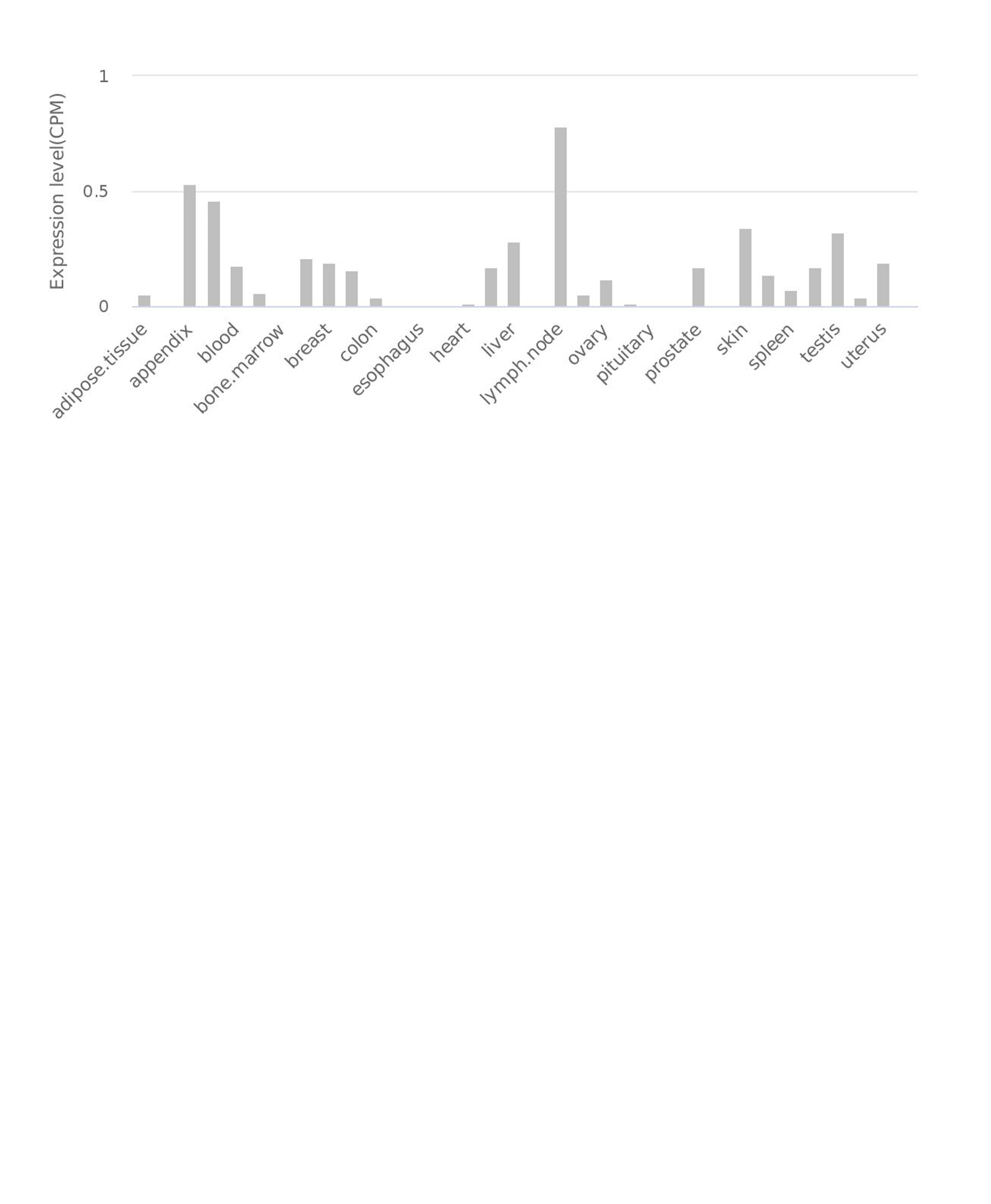

Supplement: Supplementary file 3 — Additional file 3: Fig. S3. Expression level of IL21-AS1 in human normal tissues. Expression level of IL21-AS1 was obtained from the Functional Annotation of the Mouse/Mammalian Genome database. CPM: counts per million. [file 13075_2021_2682_MOESM3_ESM.tif]
